# Mortality rates and causes of death after cardiac interventions: real-world short- and long-term insights from the Netherlands

**DOI:** 10.1007/s12471-025-02016-4

**Published:** 2026-01-21

**Authors:** Lineke Derks, Maaike M. Roefs, Gijs J. van Steenbergen, Saskia Houterman, Dennis van Veghel

**Affiliations:** 1 Netherlands Heart Registration, Utrecht, The Netherlands; 2 Department Medical Informatics (KIK), Amsterdam UMC, Amsterdam, The Netherlands; 3 Catharina Heart and Vascular Centre, Catharina Hospital, Eindhoven, The Netherlands

**Keywords:** Cardiovascular diseases, Cause of Death, Mortality, Cardiovascular Surgical Procedures, Follow-up Studies, Quality of Care, Benchmarking

## Abstract

**Objective:**

The aim of this study is to gain insight into mortality rates and causes of death after major cardiac interventions, using nationwide real-world data from the Netherlands.

**Methods:**

For this retrospective observational study, data from Statistics Netherlands and the Dutch all-payer claims database in the period 2016–2019 were used to select the intervention groups: coronary artery bypass grafting (CABG), percutaneous coronary intervention, surgical aortic valve replacement (SAVR), SAVR + CABG, mitral valve surgery, transcatheter heart valve intervention, pulmonary vein isolation and minimally-invasive maze surgery. For all interventions, survival status, date, and cause of death were retrieved. Causes of death were clustered for cardiovascular (CV) and non-CV causes by their corresponding ICD-10 code at different time intervals up to 5 years after the intervention.

**Results:**

A total of 203,001 interventions were included, and 13.7% (27,832) of the patients died during the 5‑year follow-up. Of these, 45.1% (12,560) were CV, and 54.9% (15,272) were non-CV deaths. After coronary revascularization, valve intervention, and aortic valve intervention and coronary revascularization combined, respectively, non-CV mortality increased from 14.2%, 12.9% and 20.7% at 30 days to 44.5%, 47.0% and 44.5% after 2 years. Of all deaths up to 5 years, 54.7%, 54.3% and 55.3% were non-CV.

**Conclusion:**

Initially main cause of death after cardiac intervention is CV-related. The proportion of non-CV deaths increases during follow-up, impacting survival for all patients up to 5 years after intervention. (Fig. 1)

**Supplementary Information:**

The online version of this article (10.1007/s12471-025-02016-4) contains supplementary material, which is available to authorized users.

## What’s new?

In this study, we provide a comprehensive analysis of short- and long-term mortality rates and causes of death following major cardiac interventions, based on nationwide real-world data from Statistics Netherlands and the Dutch all-payer claims database (Vektis). Our findings reveal that cardiovascular causes are the predominant contributors to early post-interventional mortality, while non-cardiovascular deaths increasingly impact long-term survival across all intervention groups.

The findings of study based on real-world data improves our understanding of the timing and nature of mortality following cardiac interventions in a nationwide perspective. It raises important questions about the continued reliance on all-cause mortality in long-term outcome evaluation, clinical decision making and benchmarking analyses. In addition, it demonstrates that data linkage between several data sources is a feasible approach to enable nationwide analyses without additional administration burden.

## Introduction

Cardiovascular diseases are the main cause of death in the United States and Europe, including the Netherlands [1–3]. In 2021, 5.29 million deaths of EU residents were registered, of which 32.4% (1.7 million) were attributed to circulatory diseases [3]. Although still substantial, cardiovascular mortality has steadily declined in recent years due to advances in medical care and innovative treatments [2, 4, 5]. Nationwide quality improvement initiatives and systematic outcome monitoring based on real-world data have also contributed to improved outcomes in cardiac care [6–9].

Survival is a key metric in clinical decision making and quality improvement programs, with all-cause mortality often used as the main outcome parameter in benchmarking [10–12]. While some randomized controlled trials have reported causes of death following cardiac interventions, such data remain scarce in real-world populations. As a result, it is often unclear whether (long-term) mortality is related to the underlying disease, the intervention itself, or other (unrelated) comorbidities. A better understanding of causes of death and their timing following cardiac interventions in a nationwide perspective could provide insight into life expectancy, thereby supporting more informed clinical decision-making and benchmarking.

Therefore, this study aims to provide a comprehensive overview of mortality rates and causes of death following the most common cardiac interventions, using nationwide real-world data from the Netherlands.

## Methods

### Patient selection and follow-up

This retrospective observational linkage study included all patients in the Netherlands who underwent one of the following procedures between 2016 and 2019: coronary revascularization (coronary artery bypass graft (CABG) or percutaneous coronary intervention (PCI)), valve intervention (surgical aortic valve replacement (SAVR), transcatheter heart valve intervention (THI; including transcatheter aortic valve implantation and MitraClips) or mitral valve surgery (MVS; replacement and repair)), combined aortic valve intervention and coronary revascularization (SAVR + CABG), and atrial fibrillation treatment (pulmonary vein isolation (PVI) or minimally-invasive maze surgery (mini-MAZE)). This selection is based on the public reporting for these surgical and cardiological intervention groups by the Netherlands Heart Registration (NHR) [13]. Per patient, the first intervention in the study period was included. Follow-up data on mortality from the intervention date through 31 December 2021 were linked. Patients with unknown survival status or cause of death were excluded.

### Endpoints

Primary outcomes were cause of death at 30-day, 1‑year, 2‑year, and up to 5 years. For each time point, only patients with complete follow-up were included. Causes of death were classified as cardiovascular (ICD-10 I00–I99) versus non-cardiovascular (all other ICD-10 codes). Cardiovascular deaths were further subdivided into cardiac (I20–I25; I30–I52) and non-cardiac (I00–I25; I26–I28; I60–I99). Further classifications, where applicable, included angina pectoris (I20), myocardial infarction (I21–I23), endocarditis (I33; I38–I39), myocarditis (I40–I41), cardiomyopathy (I42–I43), cardiac arrest (I46; I49), aortic aneurysm and dissection (ATAD, I71), and cerebrovascular accident (CVA, I63–I64). Malignancies were presented separately among non-cardiovascular causes of death when applicable.

### Data sources and linkage

Data linkage and analyses were performed within the secure environment of Statistics Netherlands (in Dutch: CBS). Linkage was done using a pseudonymized identifier (RIN) derived from the citizen service number. The nationwide all-payer claims database managed by Dutch Health Care Information Centre (Vektis), covering > 99.9% of the Dutch population, was used to select our cohort using procedure-specific healthcare activity codes (for selection, Tab S1 (Electronic Supplementary Material)) [13]. Death records were retrieved from the Personal Records Database (in Dutch: BRP), which is maintained by Statistics Netherlands, including survival status, date, and cause of death. For each deceased individual, a cause-of-death certificate (B-certificate) is completed by the attending medical practitioner (certifier). Since 2013, cause-of-death certificates have been coded automatically using the Iris international software program [14]. Causes of death are attributed to specific codes obtained from the 10th Revision of the International Statistical Classification of Diseases and Related Health Problems (ICD) of the World Health Organisation (WHO) [15, 16].

### Statistical analysis

Patient counts were presented in frequency tables. Mortality rates per intervention during follow-up were based on the number at risk for the specific follow-up time. For each intervention group, causes of death for 30-day, 1‑year, and 2‑year mortality were presented as counts with percentages. Survival curves up to 5 years per intervention were estimated using Cox regression and visualized by cardiovascular versus non-cardiovascular causes of death using the R‑package *mstate* [17]. Chi-squared tests were used to assess the equal distribution of frequencies. Due to privacy regulations of Statistics Netherlands, causes of death were not displayed when the event counts were < 11.

A *p*-value < 0.05 was considered statistically significant. All analyses were conducted using R Studio v3.6.2 (R Foundation for Statistical Computing, Vienna, Austria, www.r-project.org).

## Results

Between 2016 and 2019, 203,043 patients underwent a cardiac intervention. Complete data were available for 203,001 patients. Figure 2 shows the number of patients per intervention group. Follow-up was complete for all patients up to 2 years after intervention, and for 69,004 patients (34.0%) up to 5 years. During the study period, 13.7% (27,832) of patients died, including 12,560 (45.1%) cardiovascular deaths and 15,272 (54.9%) non-cardiovascular deaths. Cardiovascular causes of death were predominant at 30 days (85.6%, *n* = 3,859), 1 year (64.3%, *n* = 6,416), and 2 years (54.9%, *n* = 8,380) after intervention.

**Fig. 1:**
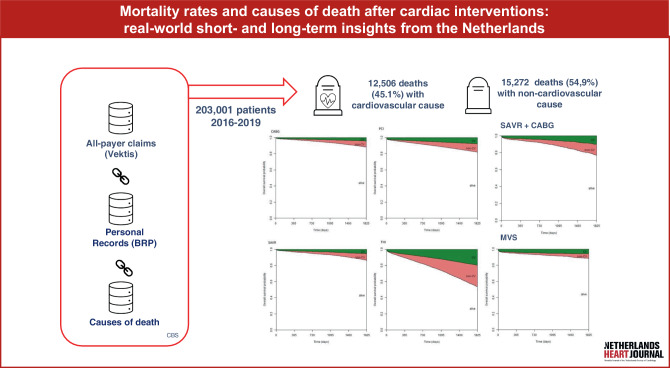
Infographic

**Fig. 2:**
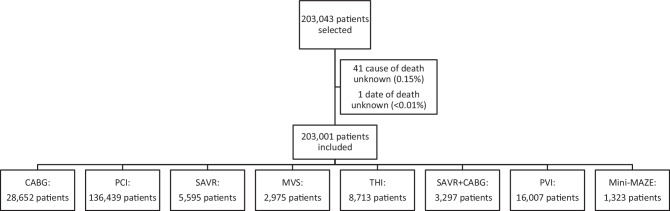
Flowchart of included patients per intervention group. (*CABG* coronary artery bypass graft, *PCI* percutaneous coronary intervention, *SAVR* surgical aortic valve replacement, *THI* transcatheter heart valve intervention, *MVS* mitral valve surgery, *PVI* pulmonary vein isolation, *mini-MAZE* minimally invasive maze surgery)

### Coronary revascularization

At 30-day follow-up, all-cause mortality after coronary revascularization (CABG and PCI) was 2.4% (*n* = 3,998), of which 85.8% (*n* = 3,429) were due to cardiovascular causes. After 1‑year, all-cause mortality increased to 5.1% (*n* = 8,447), with 64.9% (*n* = 5,482) cardiovascular and 35.1% (*n* = 2,965) non-cardiovascular mortality. After 2 years, all-cause mortality was 7.9% (*n* = 12,740), with 55.5% (*n* = 7,066) cardiovascular and 44.5% (*n* = 5,674) non-cardiovascular deaths.

**Fig. 3:**
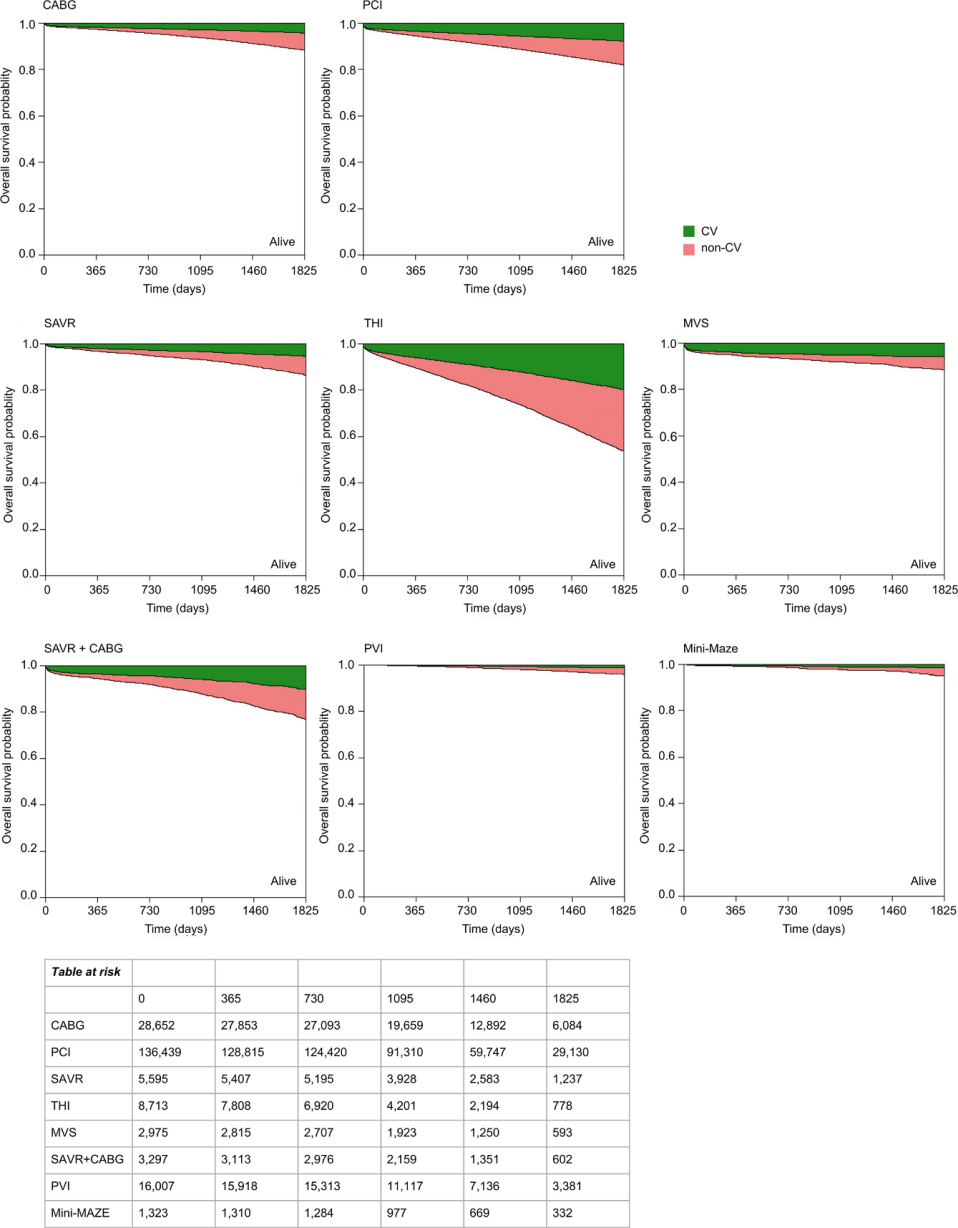
Survival curves up to 5 years after cardiac intervention, divided by proportion of cardiovascular (*CV*) and non-CV causes of death. (*CV* Cardiovascular, *CABG* coronary artery bypass graft, *PCI* percutaneous coronary intervention, *SAVR* surgical aortic valve replacement, *THI* transcatheter heart valve intervention, *MVS* mitral valve surgery, *PVI* pulmonary vein isolation, *mini-MAZE* minimally invasive maze surgery)

At 30 days after CABG, cardiovascular causes were the predominant cause of death compared to non-cardiovascular causes (87.0% (*n* = 322), *P-*value < 0.01) (Tab. 1); chronic ischaemic heart disease was a prominent cause of death (52.3%, *n* = 194) (Tab S2, Electronic Supplementary Material). In the period 30 days until 2 years after CABG, there were 906 deaths, of which 58.1% (*n* = 527) were due to non-cardiovascular causes. Non-cardiovascular deaths continued to increase up to 5 years (Fig. 3). Overall, non-cardiovascular causes accounted for 58.9% (*n* = 1,530) and malignancies for 19.0% (*n* = 493) of all causes of death after CABG (Tab S2, Electronic Supplementary Material).

**Table 1:** Cardiovascular (CV) and non-cardiovascular (Non-CV) causes of death during follow-up after coronary revascularization and valve intervention

	Coronary revascularization						Valve intervention							
	All (*n* = 165,091)		CABG (*n* = 28,652)		PCI (*n* = 136,439)		All (*n* = 17,283)		SAVR (*n* = 5,595)		THI (*n* = 8,713)		MVS (*n* = 2,975)	
	*n*	%	*n*	%	*n*	%	*n*	%	*n*	%	*n*	%	*n*	%
*30-day mortality*	3,998	2.4%	370	1.3%	3,628	2.7%	419	2.4%	71	1.3%	257	2.9%	91	3.1%
CV	3,429	85.8%	322	87.0%	3,107	85.6%	365	87.1%	60	84.5%	226	87.9%	79	86.8%
Non-CV	569	14.2%	48	13.0%	521	14.4%	54	12.9%	11	15.5%	31	12.1%	12	13.2%
*1‑year mortality*	8,447	5.1%	800	2.8%	7,647	5.6%	1,256	7.3%	188	3.4%	907	10.4%	161	5.4%
CV	5,482	64.9%	544	68.0%	4,938	64.6%	770	61.3%	118	62.8%	527	58.1%	125	77.6%
Non-CV	2,965	35.1%	256	32.0%	2,709	35.4%	486	38.7%	70	37.2%	380	41.9%	36	22.4%
*2‑year mortality*	12,740	7.9%	1,276	4.5%	11,464	8.4%	2,058	12.2%	292	5.3%	1,564	18.4%	202	7.0%
CV	7,066	55.5%	701	54.9%	6,365	55.5%	1,091	53.0%	162	55.5%	786	50.3%	143	70.8%
Non-CV	5,674	44.5%	575	45.1%	5,099	44.5%	967	47.0%	130	44.5%	778	49.7%	59	29.2%
*Overall mortality*	22,928		2,592		20,336		3,804		591		2,923		290	
CV	10,386	45.3%	1,062	41.0%	9,324	45.8%	1,738	45.7%	266	45.0%	1,304	44.6%	168	57.9%
Non-CV	12,542	54.7%	1,530	59.0%	11,012	54.2%	2,066	54.3%	325	55.0%	1,619	55.4%	122	42.1%

Counts below 11 could not be extracted, therefore numbers do not add up to 100%. Overall mortality represents all mortality during the 5‑year study period and thus also includes patients who have not reached a minimum of 5‑year follow-up

*CABG* coronary artery bypass graft, *PCI* percutaneous coronary interventions, *SAVR* surgical aortic valve replacement, *THI* transcatheter heart intervention, *MVS* mitral valve surgery

At 30 days after PCI, the cause of death was predominantly cardiovascular (85.6% (*n* = 3,107), *P-*value < 0.001) (Tab. 1), and myocardial infarction was the most prominent cause of death (55.6%, *n* = 2,018) (Tab S3, Electronic Supplementary Material). After 30 days to 2 years after PCI, there were 7,836 deaths; 58.4% (*n* = 4,578) due to cardiovascular causes and 18.2% (*n* = 1,355) due to malignancies (Tab S3, Electronic Supplementary Material). Non-cardiovascular mortality predominated up to 5 years after PCI, comprising 54.2% (*n* = 11,012) of all deaths (Fig. 3).

### Valve intervention

At 30-day follow-up, all-cause mortality in valve interventions (SAVR, THI, and MVS) was 2.4% (*n* = 419), of which 87.1% (*n* = 365) were due to cardiovascular causes. After 1 year, all-cause mortality increased to 7.3% (*n* = 1,256), with 61.3% (*n* = 770) cardiovascular and 38.7% (*n* = 486) non-cardiovascular mortality. After 2 years, all-cause mortality increased to 12.2% (*n* = 2,058), with 53.0% (*n* = 1,091) cardiovascular and 47.0% (*n* = 967) non-cardiovascular deaths.

Cardiovascular causes of death were the predominant cause of death after SAVR compared to non-cardiovascular causes at 30 days (84.5% (*n* = 60), *P-*value < 0.001) (Tab. 1). Thereafter, the proportion of non-cardiovascular increased, of the 221 deaths between 30 days until 2 years after the intervention 53.8% (*n* = 119) were due to non-cardiovascular causes (Tab. 1). The survival up to 5 years is shown in Fig. 3; 55.0% (*n* = 325) of all deaths were non-cardiovascular. More detailed information on causes of death after SAVR is provided in Tab S4 (Electronic Supplementary Material).

After THI, cardiovascular causes were the predominant cause of death compared to non-cardiovascular causes at 30 days (87.9% (*n* = 226), *P*-value < 0.001) (Tab. 1). Of the 1,307 deaths between 30 days and 2 years after THI (difference between 1,564 deaths after 2 years and 257 deaths after 30 days), only 42.8% (*n* = 560) were due to cardiovascular causes, of which 10.1% (*n* = 132) were due to heart failure (Tab S5, Electronic Supplementary Material). Of all deaths up to 5 years after THI, the proportion of non-cardiovascular causes was 55.4% (*n* = 1,619), Fig. 3 shows this trend.

After MVS, cardiovascular causes were the predominant cause of death compared to non-cardiovascular causes at 30 days (86.8% (*n* = 79), *P-*value < 0.001) (Tab. 1), of which endocarditis was one of the most prominent causes (22.0%, *n* = 20) (Tab S6, Electronic Supplementary Material). Cardiovascular causes remained the most prominent compared to non-cardiovascular causes at 1 year (77.6% (*n* = 125), *P-*value < 0.001) and 2 years (70.8% (*n* = 143), *P-*value < 0.001) (Tab. 1). Figure 3 shows the converging trend up to 5 years post-intervention. In total, 42.1% (*n* = 122) of all deaths were due to non-cardiovascular causes.

### Combined aortic valve intervention and coronary revascularization

Of the total deaths within 30 days after SAVR + CABG treatment, 79.3% (*n* = 65) died due to cardiovascular causes (Tab. 2), chronic ischemic heart disease was the most prominent cause of death (32.9%, *n* = 27) (Tab S7, Electronic Supplementary Material). Cardiovascular causes were predominant after SAVR + CABG compared to non-cardiovascular causes after 1 year (64.3% (*n* = 119), *P-*value: 0.005) but not after 2 years (54.5% (*n* = 146), *P-*value: 0.299) (Tab. 2). Non-cardiovascular causes accounted for 56.5% (*n* = 105) of the 186 deaths between 30 days and 2 years post-intervention. Figure 3 shows this trend for SAVR + CABG up to 5 years. Overall, 55.3% (*n* = 319) of all deaths were non-cardiovascular.

**Table 2:** Cardiovascular (CV) and non-cardiovascular (Non-CV) causes of death during follow-up after cardiac intervention

	Combined aortic valve intervention and coronary revascularization		Atrial fibrillation treatment			
	SAVR + CABG (*n* = 3,297)		PVI (*n* = 16,007)		Mini-MAZE (*n* = 1,323)	
	*n*	%	*n*	%	*n*	%
*30-day mortality*	82	2.5%	11	0.1%	n. a.	n. a.
CV	65	79.3%	n. a.	n. a.	n. a.	n. a.
Non-CV	17	20.7%	n. a.	n. a.	n. a.	n. a.
*1‑year mortality*	185	5.6%	89	0.6%	n. a.	n. a.
CV	119	64.3%	45	50.6%	n. a.	n. a.
Non-CV	66	35.7%	44	49.4%	n. a.	n. a.
*2‑year mortality*	268	8.3%	188	1.2%	19	1.5%
CV	146	54.5%	77	41.0%	n. a.	n. a.
Non-CV	122	44.5%	111	59.0%	n. a.	n. a.
*Overall mortality*	577		471		52	
CV	258	44.7%	160	34.0%	18	34.6%
Non-CV	319	55.3%	311	66.0%	34	65.4%

Counts below 11 could not be extracted, therefore numbers do not add up to 100%. Overall mortality represents all mortality during the 5‑year study period and thus also includes patients who have not reached a minimum of 5‑year follow-up

*CABG* coronary artery bypass graft, *SAVR* surgical aortic valve replacement, *PVI* pulmonary vein isolation, *Mini-MAZE* minimally invasive maze surgery, *n.* *a.* data not available

### Atrial fibrillation treatment

Mortality rates among patients treated with PVI or mini-MAZE were low (2-year all-cause mortality 1.2% (*n* = 188) and 1.5% (*n* = 19), respectively). One year after PVI, cardiovascular and non-cardiovascular causes of death were equal (50.6% (*n* = 45) vs. 49.4% (*n* = 44), *p*-value: 1.00) (Tab. 2). Of the 99 deaths in the following year, 33.4% were due to malignancies, resulting in non-cardiovascular causes being predominant 2 years after PVI (59.0%, (*n* = 111), *p*-value: 0.01). More detailed information on PVI and mini-MAZE can be found in Tab S8–S9 (Electronic Supplementary Material). For both PVI and mini-MAZE up to 5 years, non-cardiovascular causes are more common (66.0% (*n* = 311) and 65.5% (*n* = 34), respectively; Fig. 3).

## Discussion

Our study shows that in all cardiac intervention groups, the majority of mortality in the first 30 days after the intervention had a cardiovascular cause, while non-cardiovascular mortality predominated after 5 years in all groups except MVS. By using complete, population-wide data from all Dutch hospitals and linking procedural data to national mortality records, this study provides a unique and comprehensive overview of the shifting distribution of causes of death over time after cardiac interventions.

These findings are consistent with previous studies reporting similar shifts in causes of death following cardiac interventions. For example, Østergaard et al. observed a decrease in cardiovascular mortality from 53.8% in the first year to 32.7% 7 years post-TAVI in a study of 3,434 patients from the Danish National Registry [18]. Watanabe et al. observed that cardiac mortality accounted for only 36.6% of all mortality at a median follow-up of 5.1 years in 15,231 patients from a Japanese multicentre PCI/CABG registry, with vascular and non-cardiovascular mortality comprising 11.9% and 51.5%, respectively [19]. Similar trends have been observed for PCI, AVR, and MVS in SWEDEHEART and other cohorts [20–22]. Furthermore, neoplasms accounted for 8–20% of non-cardiovascular mortality in our study, supporting previous findings [18, 19, 21]. Together, these results highlight a consistent pattern across interventions and populations, raising important questions about the continued reliance on all-cause mortality in long-term outcome evaluation.

All-cause mortality is still often used as an outcome in (shared) clinical decision making and quality improvement programs for benchmarking analyses [10–12]. Our findings demonstrate that focusing on cardiovascular mortality alone, without increasing registration burden, may yield more meaningful insights. When benchmarking hospitals or examining regional differences based solely on all-cause mortality, analyses may unknowingly reflect factors unrelated to the disease or intervention. This could potentially discourage quality improvement efforts or lead to the implementation of unnecessary improvement strategies. Focusing on cardiovascular causes or reducing follow-up duration may provide better insight into intervention-related mortality and opportunities for improving cardiac care. Future studies should re-evaluate the role of all-cause mortality as a quality metric in registry-based quality improvement.

Providing clinicians and patients with information on cardiovascular vs. non-cardiovascular mortality could aid shared decision-making in choosing the most appropriate treatment strategy, including conservative treatment strategies. Comparing long-term survival and cause-of-death trends between cardiovascular patients and the general population could be another focus of future research, offering additional prognostic insights for both patients and caregivers.

### Limitations

While this study provides unique insights into causes of death after cardiac interventions in the Netherlands, some limitations should be noted. Firstly, interventions were included based on the nationwide all-payer claims database, and as such, data on patient and intervention characteristics and outcomes were not available. Recent successful data linkages between the Netherlands Heart Registration and Statistics Netherlands [23, 24], will help overcome this limitation in future studies. Secondly, prior studies have shown large international variation in the quality of cause-of-death data [25–27]. In the Netherlands, the cause of death is determined by a medical practitioner and submitted to Statistics Netherlands. When no autopsy is performed, misclassification is possible, although plausibility checks and follow-up by Statistics Netherlands mitigate this risk [15]. In our study, the proportion of missing cause-of-death data was very low (0.15%). Thirdly, due to privacy restrictions and possible identification of individuals, data could not be stratified into potentially interesting groups, for example, sex or age, and ICD-10 codes could not be reported at their most detailed level. Lastly, follow-up data were complete for up to 2 years post-intervention, with only a smaller subset of participants having follow-up data available up to 5 years. Although this does not prevent assessment of causes of death, the limited long-term follow-up may introduce bias if the subset with extended data is not representative of the full cohort.

## Conclusion

This study using nationwide real-world data shows that initially the main cause of death after cardiac intervention is cardiovascular related. The proportion of non-cardiovascular deaths increases up to 5 years after all major types of cardiac interventions. In addition, this study demonstrates that data linkage between several data sources is a feasible approach to enable nationwide analyses with minimized administration burden.

## Supplementary Information

Supplementary material

## Data Availability

Results based on calculations by Amsterdam UMC in project 9246 using non-public microdata from Statistics Netherlands. Under certain conditions, this microdata is accessible for statistical and scientific research. For further information: Microdata: Conducting your own research | CBS.
