# Supplementary material for: Mortality rates and causes of death after cardiac interventions: real-world short- and long-term insights from the Netherlands
Source: Neth Heart J. 2026 Jan 21;34(2):60–71. doi: 10.1007/s12471-025-02016-4 (PMC12852519; doi:10.1007/s12471-025-02016-4)
Supplement: Supplementary file 1 — Supplementary material [file 12471_2025_2016_MOESM1_ESM.docx]

**SUPPLEMENTAL MATERIAL**

**Table S1:** Selection of health care activity codes to construct intervention groups

| **Coronary artery bypass graft (CABG)** | **Health care activity code** | **Type** | **Description [in dutch]** |
| --- | --- | --- | --- |
|  | 033104 | ZA - Verrichting | Aortocoronaire bypass operatie met uitsluitend veneuze graft(s) en/of kunststof materiaal. |
|  | 033105 | ZA - Verrichting | Aortocoronaire bypass met 1 arteriële graft, inclusief eventuele veneuze graft(s) en/of kunststof materiaal. |
|  | 033106 | ZA - Verrichting | Aortocoronaire bypass operatie met 2 arteriële grafts, inclusief eventuele veneuze graft(s) en/of kunststof materiaal. |
|  | 033107 | ZA - Verrichting | Aortocoronaire bypass operatie met 3 of meer arteriële grafts, inclusief eventuele veneuze graft(s) en/of kunststof materiaal. |
|  | 033098 | ZA - Verrichting | Vervanging aorta ascendens zonder circulatiestilstand. |
|  | 979001199 | Zorgproduct | Ingewikkelde omleidingsoperatie bij een hartaandoening/longaandoening |
|  | 979001200 | Zorgproduct | Minder ingewikkelde omleidingsoperatie bij een hartaandoening/longaandoening |
|  | 979001201 | Zorgproduct | Omleidingsoperatie bij een hartaandoening/longaandoening |
|  | **Selection: Indien 1 of meer zorgactiviteitcode AND 1 verwacht zorgproduct** | | |
|  |  |  |  |
| **Percutaneous coronary intervention (PCI)** | **Health care activity code** | **Type** | **Description [in dutch]** |
|  | 033231 | ZA - Verrichting | PTCA eentak ter opheffing/verwijdering stenosen coronaire arterien. |
|  | 033232 | ZA - Verrichting | PTCA meertak of hoofdstam ter opheffing/verwijdering stenosen coronaire arterien. |
|  | 033233 | ZA - Verrichting | PTCA ter opheffing/verwijdering chronische occlusie coronaire arterien. |
|  | 033234 | ZA - Verrichting | PTCA met passage coronaire arterien graft. |
|  | 033235 | ZA -Verrichting | PTCA ter sluiting coronaire fistel. |
|  | 033238 | ZA - Verrichting | Acute PTCA ter opheffing/verwijdering stenosen coronaire. |
|  | **Selection: Indien 1 of meer zorgactiviteitcode** | | |
|  |  |  |  |
| **Surgical aortic valve replacement (SAVR)** | **Health care activity code** | **Type** | **Description [in dutch]** |
|  | 033079 | ZA -Verrichting | Hartklepvervanging, open procedure. |
|  | 190291 | ZA -Materiaal | Aortaklepprothese |
|  | 0328-23-00-2325 | Diagnose | Aortaklepvervanging (AVR) |
|  | 979001193 | Zorgproduct | Klepchirurgie \| 1 kostenunit \| Hartoperatie/hart-/longtransplantatie |
|  | **Selection: Indien ((zorgactiviteit 033079 OR zorgactiviteit 190291) AND diagnose 2325 AND product 979001193)** | | |
|  |  | | |
| **Transcatheter heart valve intervention (THI)** | **Health care activity code** | **Type** | **Description [in dutch]** |
|  | 190619 | ZA -Verrichting | Transkatheter hartklep |
|  | 033247 | ZA -Verrichting | Percutane hartklep implantatie |
|  | **Selection: Indien 1 of meer zorgactiviteitcode** | | |
|  |  |  |  |
| **Mitral valve surgery (MVS; including replacement and repair)** | **Health care activity code** | **Type** | **Description [in dutch]** |
|  | 033079 | ZA -Verrichting | Hartklepvervanging, open procedure. |
|  | 033078 | ZA -Verrichting | Hartklepplastiek, open procedure. |
|  | 033085 | ZA -Verrichting | Thoracoscopische plastiek of vervanging van hartklep |
|  | 190292 | ZA -Materiaal | Mitralisklepprothese. |
|  | 190624 | ZA -Materiaal | Hartklepring. |
|  | 0328-23-00-2335 | Diagnose | Mitraalklepvervanging (MVR) |
|  | 0328-24-00-2405 | Diagnose | Mitralisklep plastiek (MPL) |
|  | 979001193 | Zorgproduct | Klepchirurgie \| 1 kostenunit \| Hartoperatie/hart-/longtransplantatie |
|  | 979001192 | Zorgproduct | Klepchirurgie \| 2 kostenunits \| Hartoperatie/hart-/longtransplantatie |
|  | **Selection: Indien 1 of meer zorgactiviteitcode AND 1 verwachte diagnose AND 1 verwacht zorgproduct is vastgelegd** | | |
|  |  |  |  |
| **SAVR + CABG** | **Health care activity code** | **Type** | **Description [in dutch]** |
|  | 033079 | ZA -Verrichting | Hartklepvervanging, open procedure. |
|  | 190291 | ZA -Materiaal | Aortaklepprothese |
|  | 033104 | ZA -Verrichting | Aortocoronaire bypass operatie met uitsluitend veneuze graft(s) en/of kunststof materiaal. |
|  | 033105 | ZA -Verrichting | Aortocoronaire bypass met 1 arteriële graft, inclusief eventuele veneuze graft(s) en/of kunststof materiaal. |
|  | 033106 | ZA -Verrichting | Aortocoronaire bypass operatie met 2 arteriële grafts, inclusief eventuele veneuze graft(s) en/of kunststof materiaal. |
|  | 033107 | ZA -Verrichting | Aortocoronaire bypass operatie met 3 of meer arteriële grafts, inclusief eventuele veneuze graft(s) en/of kunststof materiaal. |
|  | 033098 | ZA -Verrichting | Vervanging aorta ascendens zonder circulatiestilstand. |
|  | 0328-24-00-2425 | Diagnose | CABG (1 art) + AVR |
|  | 0328-25-00-2570 | Diagnose | CABG (2 art) + AVR |
|  | 979001192 | Zorgproduct | Klepchirurgie \| 2 kostenunits \| Hartoperatie/hart-/longtransplantatie |
|  | **Selection: Indien 1 of meer zorgactiviteitcode AND 1 verwachte diagnose AND 1 verwacht zorgproduct is vastgelegd** | | |
|  |  | | |
| **Pulmonary vein isolation (PVI) for atrial fibrillation** | **Health care activity code** | **Type** | **Description [in dutch]** |
|  | 032946 | ZA -Verrichting | Catheterablatie linker atrium. |
|  | **Selection: Indien zorgactiviteit 032946** | | |
|  |  |  |  |
| **Minimally invasive maze surgery (mini-MAZE) for atrial fibrillation** | **Health care activity code** | **Type** | **Description [in dutch]** |
|  | 033077 | ZA -Verrichting | Endoscopische MAZE-procedure. |
|  | 0328-25-00-2525 | Diagnose | Maze procedure |
|  | 979001195 | Zorgproduct | Oper myo-/peri-/endocard \| Meest complex \| Hartoperatie/hart-/longtransplantatie |
|  | **Selection: Indien ( zorgactiviteit 033077 AND diagnose 2525 AND zorgproduct 979001195 )** | | |

**Coronary artery revascularization**

| **Table S2:** Causes of death by rate - CABG | | | | | | | | | | |
| --- | --- | --- | --- | --- | --- | --- | --- | --- | --- | --- |
| **Cause of death** |  |  | **30-day mortality (N=370)** | | **1-year mortality (N=800)** | | **2-year mortality (N=1276)** | | **overall mortality (N=2592)** | |
|  |  |  | **n** | **%** | **n** | **%** | **n** | **%** | **n** | **%** |
| Non-cardiovascular | Total |  | 48 | 12.9 | 256 | 32.0 | 575 | 45.1 | 1530 | 58.9 |
|  | Malignancy |  |  |  | 73 | 9.1 | 195 | 15.3 | 493 | 19.0 |
|  | Other |  |  |  | 183 | 22.9 | 380 | 29.8 | 1037 | 39.9 |
| Cardiovascular | Total |  | 322 | 86.8 | 544 | 67.9 | 701 | 54.9 | 1062 | 40.9 |
|  | Non-cardiac | Total | 20 | 5.4 | 70 | 8.8 | 118 | 9.2 | 229 | 8.8 |
|  |  | ATAD |  |  |  |  | 10 | 0.8 | 19 | 0.7 |
|  |  | CVA | 10 | 2.7 |  |  | 37 | 2.9 | 76 | 2.9 |
|  |  | Other non-cardiac |  |  | 40 | 5.0 | 71 | 5.6 | 134 | 5.2 |
|  | Cardiac | Total | 302 | 81.4 | 474 | 59.1 | 583 | 45.7 | 833 | 32.0 |
|  |  | Chronic ischaemic heart disease | 194 | 52.3 | 293 | 36.6 | 331 | 25.9 | 415 | 16.0 |
|  |  | Heart failure |  |  | 40 | 5.0 | 61 | 4.8 | 129 | 5.0 |
|  |  | Myocardial infarction | 78 | 21.0 | 105 | 13.1 | 128 | 10.0 | 180 | 6.9 |
|  |  | Endocarditis / Cardiac arrest / Cardiomyopathy |  |  | 13 | 1.6 | 27 | 2.1 | 44 | 1.7 |
|  |  | Other cardiac | 13 | 3.5 | 23 | 2.9 | 36 | 2.8 | 65 | 2.5 |

Counts below 11 are not shown, therefore numbers do not add up to 100%. Patients with unknown cause of death are included in the calculation of the (sub)total percentages..
CABG: coronary artery bypass graft, ATAD: acute type A dissection, CVA: cerebral vascular incident.

| **Table S3:** Causes of death by rate - PCI | | | | | | | | | | |
| --- | --- | --- | --- | --- | --- | --- | --- | --- | --- | --- |
| **Cause of death** |  |  | **30-day mortality (N=3628)** | | **1-year mortality (N=7647)** | | **2-year mortality (N=11464)** | | **overall mortality (N=20336)** | |
|  |  |  | **n** | **%** | **n** | **%** | **n** | **%** | **n** | **%** |
| Non-cardiovascular | Total |  | 521 | 14.4 | 2709 | 35.4 | 5099 | 44.5 | 11012 | 54.2 |
|  | Malignancy |  | 72 | 2.0 | 692 | 9.0 | 1427 | 12.4 | 2995 | 14.7 |
|  | Other |  | 449 | 12.4 | 2017 | 26.4 | 3672 | 32.0 | 8017 | 39.4 |
| Cardiovascular | Total |  | 3107 | 85.6 | 4938 | 64.6 | 6365 | 55.5 | 9324 | 45.8 |
|  | Non-cardiac | Total | 129 | 3.6 | 475 | 6.2 | 808 | 7.0 | 1451 | 7.1 |
|  |  | ATAD | 25 | 0.7 | 52 | 0.7 | 92 | 0.8 | 158 | 0.8 |
|  |  | CVA | 23 | 0.6 | 110 | 1.4 | 205 | 1.8 | 411 | 2.0 |
|  |  | Other non-cardiac | 81 | 2.2 | 313 | 4.1 | 511 | 4.5 | 882 | 4.3 |
|  | Cardiac | Total | 2974 | 82.0 | 4454 | 58.2 | 5543 | 48.4 | 7843 | 38.6 |
|  |  | Cardiac arrest | 111 | 3.1 | 150 | 2.0 | 188 | 1.6 | 267 | 1.3 |
|  |  | Cardiomyopathy |  |  | 37 | 0.5 | 57 | 0.5 | 92 | 0.5 |
|  |  | Chronic ischaemic heart disease | 559 | 15.4 | 1062 | 13.9 | 1355 | 11.8 | 2009 | 9.9 |
|  |  | Endocarditis |  |  | 66 | 0.9 | 105 | 0.9 | 166 | 0.8 |
|  |  | Heart failure | 92 | 2.5 | 409 | 5.3 | 715 | 6.2 | 1386 | 6.8 |
|  |  | Myocardial infarction | 2018 | 55.6 | 2332 | 30.5 | 2565 | 22.4 | 3020 | 14.9 |
|  |  | Other cardiac | 164 | 4.5 | 398 | 5.2 | 558 | 4.9 | 903 | 4.4 |

Counts below 11 are not shown therefore numbers do not add up to 100%.
PCI: percutaneous coronary interventions, ATAD: acute type A dissection, CVA: cerebral vascular incident.

**Valve intervention**

| **Table S4:** Causes of death by rate - SAVR | | | | | | | | | | |
| --- | --- | --- | --- | --- | --- | --- | --- | --- | --- | --- |
| **Cause of death** |  |  | **30-day mortality (N=71)** | | **1-year mortality (N=188)** | | **2-year mortality (N=292)** | | **overall mortality (N=591)** | |
|  |  |  | **n** | **%** | **n** | **%** | **n** | **%** | **n** | **%** |
| Non-cardiovascular | Total |  | 11 | 15.5 | 70 | 37.2 | 130 | 44.2 | 325 | 54.8 |
|  | Malignancy |  |  |  | 28 | 14.9 | 52 | 17.7 | 121 | 20.4 |
|  | Other |  |  |  | 42 | 22.3 | 78 | 26.5 | 204 | 34.4 |
| Cardiovascular | Total |  | 60 | 84.5 | 118 | 62.8 | 162 | 55.1 | 266 | 44.9 |
|  | Non-cardiac | Total |  |  | 16 | 8.5 | 27 | 9.2 | 54 | 9.1 |
|  |  | ATAD |  |  |  |  |  |  |  |  |
|  |  | CVA |  |  |  |  |  |  |  |  |
|  |  | Other non-cardiac |  |  |  |  | 17 | 5.8 | 34 | 5.7 |
|  | Cardiac | Total | 57 | 80.3 | 102 | 54.3 | 135 | 45.9 | 212 | 35.8 |
|  |  | Endocarditis |  |  | 29 | 15.4 | 36 | 12.2 | 53 | 8.9 |
|  |  | Cardiac arrest / Cardiomyopathy / Chronic ischaemic heart disease / Heart failure / Myocardial infarction |  |  | 16 | 8.5 | 31 | 12.4 | 71 | 12.0 |
|  |  | Other cardiac | 39 | 54.9 | 57 | 30.3 | 68 | 23.1 | 88 | 14.8 |

Counts below 11 are not shown therefore numbers do not add up to 100%. Patients with unknown cause of death are included in the calculation of the (sub)total percentages..
SAVR: surgical aortic valve replacement, ATAD: acute type A dissection, CVA: cerebral vascular incident.

| **Table S5:** Causes of death by rate - THI | | | | | | | | | | |
| --- | --- | --- | --- | --- | --- | --- | --- | --- | --- | --- |
| **Cause of death** |  |  | **30-day mortality (N=257)** | | **1-year mortality (N=907)** | | **2-year mortality (N=1564)** | | **overall mortality (N=2923)** | |
|  |  |  | **n** | **%** | **n** | **%** | **n** | **%** | **n** | **%** |
| Non-cardiovascular | Total |  | 31 | 12.1 | 380 | 41.8 | 778 | 49.7 | 1619 | 55.3 |
|  | Malignancy |  |  |  | 91 | 10.0 | 199 | 12.7 | 411 | 14.0 |
|  | Other |  |  |  | 289 | 31.8 | 579 | 37.0 | 1208 | 41.3 |
| Cardiovascular | Total |  | 226 | 87.9 | 527 | 58.0 | 786 | 50.2 | 1304 | 44.6 |
|  | Non-cardiac | Total | 20 | 7.8 | 82 | 9.0 | 163 | 10.4 | 266 | 9.1 |
|  |  | ATAD |  |  | 10 | 1.1 | 20 | 1.3 | 25 | 0.9 |
|  |  | CVA |  |  | 26 | 2.9 | 51 | 3.3 | 86 | 2.9 |
|  |  | Other non-cardiac | 10 | 3.9 | 46 | 5.1 | 92 | 5.9 | 155 | 5.3 |
|  | Cardiac | Total | 206 | 80.2 | 445 | 49.0 | 623 | 39.8 | 1038 | 35.5 |
|  |  | Chronic ischaemic heart disease |  |  | 33 | 3.6 | 45 | 2.9 | 109 | 3.7 |
|  |  | Endocarditis | 12 | 4.7 | 45 | 5.0 | 65 | 4.2 | 100 | 3.4 |
|  |  | Heart failure | 13 | 5.1 | 76 | 8.4 | 145 | 9.3 | 301 | 10.3 |
|  |  | Myocardial infarction / Cardiac arrest / Cardiomyopathy |  |  | 41 | 4.5 | 64 | 4.1 | 113 | 3.9 |
|  |  | Other cardiac | 161 | 62.6 | 250 | 27.5 | 304 | 19.4 | 415 | 14.2 |

Counts below 11 are not shown therefore numbers do not add up to 100%. Patients with unknown cause of death are included in the calculation of the (sub)total percentages..
THI: transcatheter heart intervention, ATAD: acute type A dissection, CVA: cerebral vascular incident.

| **Table S6:** Causes of death by rate - MVS | | | | | | | | | | |
| --- | --- | --- | --- | --- | --- | --- | --- | --- | --- | --- |
| **Cause of death** |  |  | **30-day mortality (N=91)** | | **1-year mortality (N=161)** | | **2-year mortality (N=202)** | | **overall mortality (N=290)** | |
|  |  |  | **n** | **%** | **n** | **%** | **n** | **%** | **n** | **%** |
| Non-cardiovascular | Total |  | 12 | 13.2 | 36 | 22.4 | 59 | 29.2 | 122 | 42.1 |
|  | Malignancy |  |  |  |  |  | 13 | 6.4 | 24 | 8.3 |
|  | Other |  |  |  |  |  | 46 | 22.8 | 98 | 33.8 |
| Cardiovascular | Total |  | 79 | 86.8 | 125 | 77.6 | 143 | 70.8 | 168 | 57.9 |
|  | Non-cardiac |  | 16 | 17.9 | 27 | 16.8 | 34 | 16.8 | 44 | 15.2 |
|  | Cardiac | Total | 63 | 69.2 | 98 | 60.9 | 109 | 54.0 | 124 | 42.8 |
|  |  | Endocarditis | 20 | 22.0 | 32 | 19.9 | 36 | 17.8 | 39 | 13.4 |
|  |  | Myocardial infarction | 15 | 16.5 | 18 | 11.2 | 19 | 9.4 | 20 | 6.9 |
|  |  | Heart failure |  |  |  |  | 10 | 5.0 | 16 | 5.5 |
|  |  | Heart failure / Cardiac arrest / Cardiomyopathy / Chronic ischaemic heart disease |  |  |  |  | 20 | 9.9 | 29 | 10.0 |
|  |  | Other cardiac | 23 | 25.3 | 32 | 19.9 | 34 | 16.8 | 36 | 12.4 |

Counts below 11 are not shown therefore numbers do not add up to 100%.
MVS: mitral valve surgery, ATAD: acute type A dissection, CVA: cerebral vascular incident.

| **Other types of intervention**  **Table S7:** Causes of death by rate - SAVR+CABG | | | | | | | | | | |
| --- | --- | --- | --- | --- | --- | --- | --- | --- | --- | --- |
| **Cause of death** |  |  | **30-day mortality (N=82)** | | **1-year mortality (N=185)** | | **2-year mortality (N=268)** | | **overall mortality (N=577)** | |
|  |  |  | **n** | **%** | **n** | **%** | **n** | **%** | **n** | **%** |
| Non-cardiovascular | Total |  | 17 | 20.7 | 66 | 35.7 | 122 | 45.5 | 319 | 55.3 |
|  | Malignancy |  |  |  | 15 | 8.1 | 33 | 12.3 | 95 | 16.5 |
|  | Other |  | 17 | 20.7 | 51 | 27.6 | 89 | 33.2 | 224 | 38.8 |
| Cardiovascular | Total |  | 65 | 79.3 | 119 | 64.3 | 146 | 54.5 | 258 | 44.7 |
|  | Non-cardiac | Total |  |  | 16 | 8.6 | 23 | 8.6 | 58 | 10.1 |
|  |  | ATAD |  |  |  |  |  |  |  |  |
|  |  | CVA |  |  |  |  |  |  |  |  |
|  |  | Other non-cardiac |  |  | 11 | 5.9 | 14 | 5.2 | 34 | 5.9 |
|  | Cardiac | Total | 61 | 74.4 | 103 | 55.7 | 123 | 45.9 | 200 | 34.7 |
|  |  | Chronic ischaemic heart disease | 27 | 32.9 | 41 | 22.2 | 43 | 16.0 | 54 | 9.4 |
|  |  | Endocarditis |  |  | 10 | 5.4 | 18 | 6.7 | 27 | 4.7 |
|  |  | Cardiac arrest / Heart failure / Myocardial infarction |  |  | 16 | 8.6 | 24 | 9.0 | 64 | 11.1 |
|  |  | Other cardiac | 24 | 29.3 | 36 | 19.5 | 38 | 14.2 | 55 | 9.5 |

Counts below 11 are not shown therefore numbers do not add up to 100%.
SAVR: surgical aortic valve replacement, CABG: coronary artery bypass graft, ATAD: acute type A dissection, CVA: cerebral vascular incident.

| **Table S8:** Causes of death by rate - PVI | | | | | | | | |
| --- | --- | --- | --- | --- | --- | --- | --- | --- |
| **Cause of death** |  |  | **1-year mortality (N=89)** | | **2-year mortality (N=188)** | | **overall mortality (N=471)** | |
|  |  |  | **n** | **%** | **n** | **%** | **n** | **%** |
| Non-cardiovascular | Total |  | 44 | 49.4 | 111 | 59.0 | 311 | 66.0 |
|  | Malignancy |  | 15 | 16.9 | 48 | 25.5 | 127 | 27.0 |
|  | Other |  | 29 | 32.6 | 63 | 33.5 | 184 | 39.1 |
| Cardiovascular | Total |  | 45 | 50.6 | 77 | 41.0 | 160 | 34.0 |
|  | Non-cardiac |  | 11 | 12.4 | 17 | 9.0 | 39 | 8.3 |
|  | Cardiac | Total | 34 | 38.2 | 60 | 31.9 | 121 | 25.7 |
|  |  | Heart failure | 10 | 11.2 | 10 | 5.3 | 22 | 4.7 |
|  |  | Cardiac arrest / Cardiomyopathy / Chronic ischaemic heart disease / Endocarditis / Myocardial infarction | 13 | 14.6 | 26 | 13.8 | 61 | 13.0 |
|  |  | Other cardiac | 11 | 12.4 | 24 | 12.8 | 38 | 8.1 |

Counts below 11 are not shown therefore numbers do not add up to 100%.
PVI: pulmonary vein isolation

| **Table S9:** Causes of death by rate – mini-MAZE | | |  |
| --- | --- | --- | --- |
| **Cause of death** | **overall mortality (N=52)** | |  |
|  | **n** | **%** |  |
| Non-cardiovascular | 34 | 65.4 |  |
| Cardiovascular | 18 | 34.6 |  |

Mini-maze: minimally invasive maze surgery
